# Evaluating Community Awareness of Osteopathic Manipulative Medicine and New Educational Programs in Fresno County, United States: A Cross-Sectional Survey

**DOI:** 10.7759/cureus.96348

**Published:** 2025-11-07

**Authors:** Layla Mazdeyasnan, Zian Shabbir, Jared Ham-Ying

**Affiliations:** 1 Research, California Health Sciences University, Clovis, USA; 2 Osteopathic Medicine, California Health Sciences University, Clovis, USA

**Keywords:** awareness, chsu-com, education, fresno, manipulative, medical, medicine, omm, osteopathic, public

## Abstract

Introduction

Osteopathic manipulative medicine (OMM) is a patient-centered, evidence-based approach emphasizing the body's ability to self-heal and the interrelationship between structure and function. Despite its established history and growing recognition, public awareness of OMM and the role of Doctor of Osteopathic Medicine (DOs) remains limited, particularly in underserved regions such as Fresno County, California. With the recent establishment of California Health Sciences University College of Osteopathic Medicine (CHSU-COM) in Clovis, understanding local awareness and perceptions of osteopathic medicine is increasingly important.

Methods

A cross-sectional survey consisting of 10 Likert-scale questions was distributed in person through a QR code at five public and educational locations throughout Fresno County. Eligible participants were adults (18+) residing in Fresno County with current or past medical conditions requiring treatment. Individuals affiliated with CHSU-COM were excluded from the survey. After data collection, 182 valid responses were analyzed using Google Forms to assess knowledge, perception, and openness toward osteopathic medicine and the presence of a local osteopathic medical school.

Results

Survey results revealed a substantial gap in awareness of osteopathic medicine, with 72% of respondents reporting being unaware of its distinction from allopathic medicine. However, over 83% expressed openness to receiving osteopathic care, and 96% agreed that treating the whole person is important. Additionally, 91% believed an osteopathic medical school would positively impact the region, yet 56% were unaware of CHSU-COM's existence. While respondents supported the holistic principles of osteopathy, nearly half (45%) remained neutral about the similarity of DO and MD training, suggesting uncertainty about osteopathic education.

Discussion

Our findings highlight a lack of awareness and a strong receptivity to osteopathic principles among Fresno County residents. Despite limited OMM and DO training knowledge, community members demonstrated interest in osteopathic care and acknowledged the potential value of a local osteopathic medical school. This discrepancy indicates an opportunity for targeted educational campaigns and community outreach to bridge the knowledge gap and integrate osteopathic care into regional healthcare systems.

Conclusion

There is a significant opportunity to enhance awareness and utilization of osteopathic medicine in Fresno County. While current awareness remains low, the community's openness to holistic care and CHSU-COM is promising for future engagement. Strategic education and outreach programs are essential to expanding recognition of osteopathy and addressing healthcare disparities in underserved regions such as California's Central Valley.

## Introduction

Osteopathic manipulative medicine (OMM) is an evolving field within healthcare that is gaining recognition as an evidence-based therapy aimed at improving patient well-being through hands-on manipulation of body structures [1,2]. In the United States, medical licensure is granted to Doctors of Osteopathic Medicine (DO) and Medical Doctors (MD), also called allopathic medicine. In addition to the standard medical licensure requirements, DOs receive specialized training in OMM as part of their medical curriculum [3]. OMM is a patient-centered approach that combines osteopathic philosophy, structural diagnosis, and a variety of hands-on techniques known as osteopathic manipulative treatment (OMT) to prevent, diagnose, and treat health issues [2]. These unique techniques enhance systemic balance and are increasingly integrated into osteopathic medical education [3]. Despite their demonstrated benefits, many individuals remain either unaware of or misinformed about the therapeutic potential of OMT [3].

Andrew Taylor Still, MD, founded osteopathy based on the belief that the body can heal itself and function as an integrated whole. He advocated for a patient-centered approach, where healthcare providers focus on treating the person as a whole rather than just treating the disease [3]. Central to his philosophy are the four key tenets of osteopathy: (1) The body is a unit; the person is a unit of the mind, body, and spirit; (2) The body is capable of self-regulation, self-healing and health maintenance; (3) Structure and function are reciprocally interrelated; and (4) Rational treatment is based upon an understanding of the basic principles of body unity, self-regulation, and the interrelationship of structure and function [2]. These principles remain fundamental to osteopathic practice today.

The musculoskeletal system plays a central role in OMM, as it addresses a wide range of neuromuscular conditions, including headaches, back, shoulder, and other joint pain, representing a significant disease burden for many Americans [1]. Although numerous studies have shown that OMT is an effective treatment for these disorders, it remains an underutilized resource in medical care [1]. Over the past 150 years, the osteopathic medical profession has experienced remarkable growth, making up about 11% of all physicians and 28% of all medical students in the United States. An increasing number of prospective physicians are choosing to become DOs, with nearly 40,000 medical students enrolled in 42 colleges of osteopathic medicine [4]. California Health Sciences University-College of Osteopathic Medicine (CHSU-COM) is the Central Valley's only osteopathic medical school. In Clovis, California, CHSU-COM welcomed its inaugural class in July 2020. To combat the ongoing physician shortage in the region, CHSU-COM is dedicated to training future doctors who are more likely to stay and practice locally. The university prepares its students to deliver high-quality, compassionate care to the diverse and underserved communities in the Central Valley.

A recent study found strong interest among MD students in an OMT elective, suggesting the value of integrating OMT-specific theoretical and practical training into medical curricula for both students and residents [5]. This aligns with growing evidence for OMM's clinical benefits, backed by evidence-based meta-analysis of 55 trials, which demonstrated that OMT is effective for acute and chronic non-specific low back pain, neck pain, and chronic non-cancer pain [6]. Expanding access to OMT education could also help address broader awareness gaps. Prior research has shown that increasing public understanding of osteopathic medicine reduces barriers to using osteopathic physicians, ultimately leading to improved health outcomes. Awareness is most strongly associated with higher education, older age, and residence in the Midwest [7]. Lack of understanding of the growing field of osteopathy and its added benefits can impact patient care by limiting access to effective, non-invasive treatment options. Increasing public awareness and education about OMM could help integrate this valuable approach into mainstream care. Moreover, by addressing misconceptions and increasing knowledge, OMM could be better utilized to improve patient outcomes and promote holistic healing [8].

Study purpose

Our survey aims to assess the current level of awareness regarding Osteopathic Medicine within Fresno County. This study aimed to assess the level of community awareness, perceptions, and openness toward osteopathic medicine among residents of Fresno County, California. Secondary objectives included evaluating public recognition of CHSU-COM and determining attitudes toward its potential impact on local healthcare. Despite OMM's extensive history and unique patient-centered approach, osteopathy remains underappreciated and underutilized in medicine [2]. This underutilization persists despite growing evidence of its effectiveness in enhancing patient outcomes and its comprehensive focus on treating the whole person, not just the symptoms [2,8].

A PubMed search uncovered limited articles on Osteopathic Medicine in Fresno County or the Central Valley of California. Notably, no studies specifically addressed awareness of osteopathy, its principles, or the region's emerging educational institutions. This highlights a significant gap in the research, particularly the absence of studies examining the low level of awareness of Osteopathic Medicine in Fresno County. This gap is especially concerning given that Fresno County is a medically underserved area with a critical need for additional healthcare providers. According to the University of California, San Francisco, the San Joaquin Valley is one of California's fastest-growing, most impoverished, and least healthy regions [9]. This region has the lowest number of doctors, nurses, and nurse practitioners per 100,000 residents compared to any other region in the state. Compounding the issue, approximately 30% of this already limited healthcare workforce is approaching retirement age [9].

In 1962, Proposition 22 eliminated the legal practice of osteopathic medicine in California, with the California Medical Association offering DOs the option to convert their degrees to MDs for a fee. After legal challenges, the California Supreme Court ruled in 1974 that DO licensure must resume, leading to the reestablishment of osteopathic medical education in the state. New schools followed, including the College of Osteopathic Medicine of the Pacific (1978), Touro University California (1997), and California Health Sciences University College of Osteopathic Medicine (2020), reflecting the growing role of osteopathic medicine in California [10].

To address the physician shortage and in California, CHSU-COM opened as California's third osteopathic medical school, the first within the Central Valley. CHSU-COM's vision involves "enhancing the wellness of our community by educating healthcare professionals dedicated to providing collaborative care for the Central Valley." Given the school's novelty, local Central Valley residents may not be aware of the new medical school in the area. Previous research conducted by CHSU-COM faculty aimed to promote osteopathic awareness by surveying medical students enrolled in a PREP program in 2019 [11]. The survey asked questions including "I am familiar with osteopathic medicine" and "I understand the difference between osteopathic and allopathic doctors" [11]. However, this study excluded the broader public from its survey. Similarly, another study surveyed employees' perspectives and knowledge related to osteopathic principles at multiple osteopathic campuses [12]. This study did not ascertain the public perception, and bias was introduced, given the institutional employment status of the participants.

## Materials and methods

This cross-sectional, survey-based study was designed to assess awareness and understanding of OMM among community members of Fresno County, California. A different study also utilized a cross-sectional survey approach to quantify perceptions and knowledge of osteopathic medicine in immigrants in New York City [13]. Similarly, Fresno County was chosen as the study site due to its diverse population and representation of both urban and rural communities within the Central Valley. Data collection took place between October 2024 and March 2025.

After receiving IRB approval, the study authors developed a survey consisting of 10 Likert-scale questions to assess community awareness and understanding of OMM in Fresno, California. Questions were designed to capture participants' awareness of OMM and their knowledge of its role in healthcare. Survey items were written at an "elementary" reading level to ensure understanding and clarity.

The final survey was administered electronically through Google Forms, automatically generating descriptive statistics and pie chart summaries of each response. This platform was chosen for its user-friendly interface, widespread accessibility, and ability to preserve participant anonymity.

To increase accessibility and participation, the survey was linked to a Quick Response (QR) code that could be scanned using a mobile device. Recruitment was conducted in person at multiple community locations across Fresno County, including California State University, Fresno, Clovis Community College, Todd Beamer Park, Woodward Park, and Sierra Bicentennial Park. These locations were selected to capture a broad demographic range, including students, families, and community members in public and non-profit entities. Participants were also informed that the survey was voluntary and anonymous. A total of 200 individuals completed the study.

Inclusion criteria required participants to be 18 years or older, with age verified through a valid driver's license. To maintain Health Insurance Portability and Accountability Act (HIPAA) compliance, no personal identifiers were collected. Participants were required to reside in Fresno County, California, and have a current or past medical condition requiring treatment. If participants met these criteria, they were asked whether a DO had ever treated them. However, this question was for informational purposes and not part of the inclusion criteria. Additionally, participants had to communicate in English and understand the survey to complete it. This study did not restrict participation based on gender.

Exclusion criteria included individuals associated with CHSU-COM, such as current or former students, employees, or faculty. Individuals with no current or past medical condition requiring treatment were also excluded, as the study targeted those with prior exposure to medical treatment.

**Figure 1:**
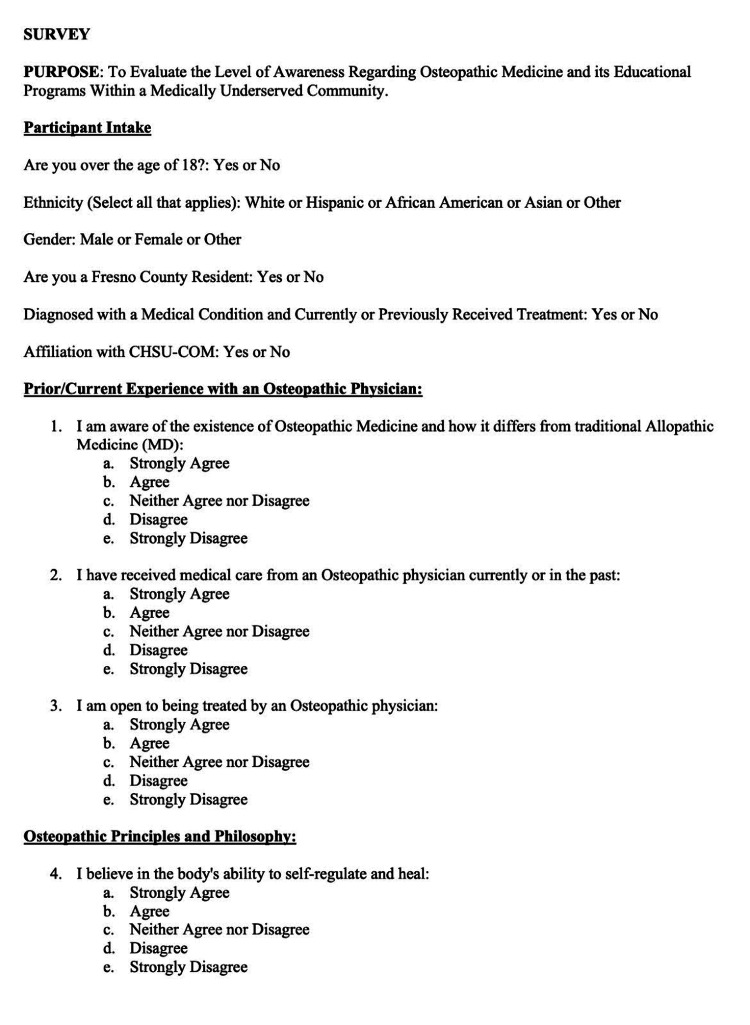
Survey Part 1

**Figure 2:**
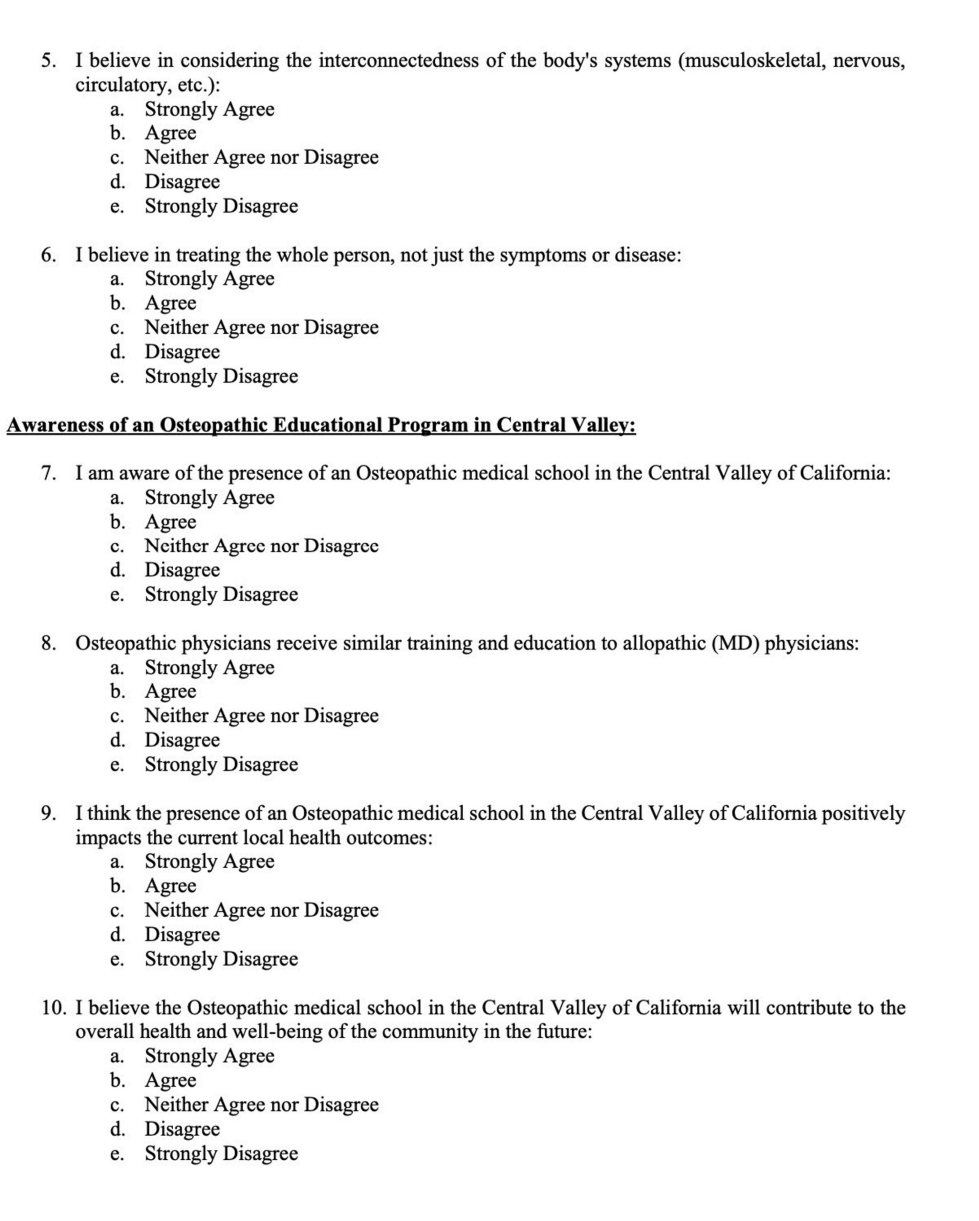
Survey Part 2

The survey was carefully worded to effectively communicate the research objectives while maintaining simplicity for the target audience (Figures 1-2).

Although the authors acknowledged the need for a certain literacy level, the survey's language was kept as straightforward to avoid altering the focus of the study. All collected data was stored securely on a password-protected laptop, and analysis was conducted using the pie charts generated by Google Forms. These charts helped categorize responses according to the Likert scale to assess the awareness level of osteopathy in the Fresno community. No statistical analysis was conducted beyond the descriptive summaries of the Likert-scale responses. All tools used in this study, including questionnaires and bar graphs, were free. We note that this study does not strictly follow Strengthening the Reporting of Observational Studies in Epidemiology (STROBE) guidelines, as it was designed as a descriptive, cross-sectional survey to assess community awareness rather than to test hypotheses or establish causal relationships.

## Results

**Figure 3:**
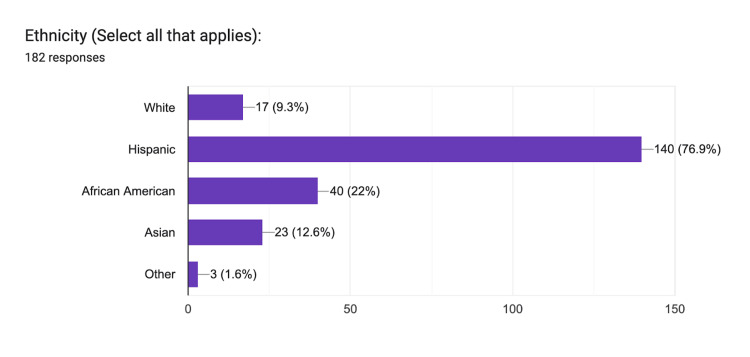
Ethnicity

For our survey, a total of 200 responses were collected. The survey results indicate that all respondents confirmed they were over the age of 18, although age was not quantified. Most respondents (n=198) were residents of Fresno County. The two responders who were not were excluded. Additionally, 93% (n=185) reported having been diagnosed with a medical condition and currently or previously receiving treatment. The 15 who reported no prior illness were excluded. Coincidentally, the two who were excluded for not being Fresno County residents were also included in this category. Finally, when evaluating respondents' affiliation with CHSU-COM, 98% reported no association with the institution. However, the four responders who indicated an affiliation were excluded, one of whom had already met the criteria for exclusion. In total, 18 surveys were excluded, with the final count of viable surveys at n=182. Regarding ethnicity, the majority identified as Hispanic individuals (77%, n=140), followed by African American individuals (22%, n=40), Asian individuals (13%, n=23), White individuals (9%, n=17), and Other individuals (2%, n=3) (Figure 3). Differences among ethnicities reflected the region's predominant population.

**Figure 4:**
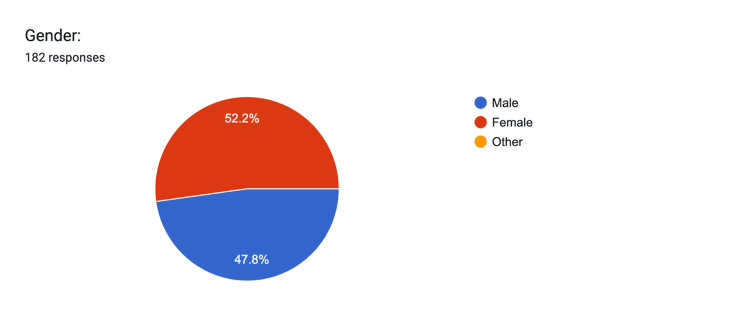
Gender

In terms of gender distribution, 52% (n=95) identified as female, while 48% (n=87) identified as male, and 0% (n=0) identified as other (Figure 4). There were no differences among genders.

**Figure 5:**
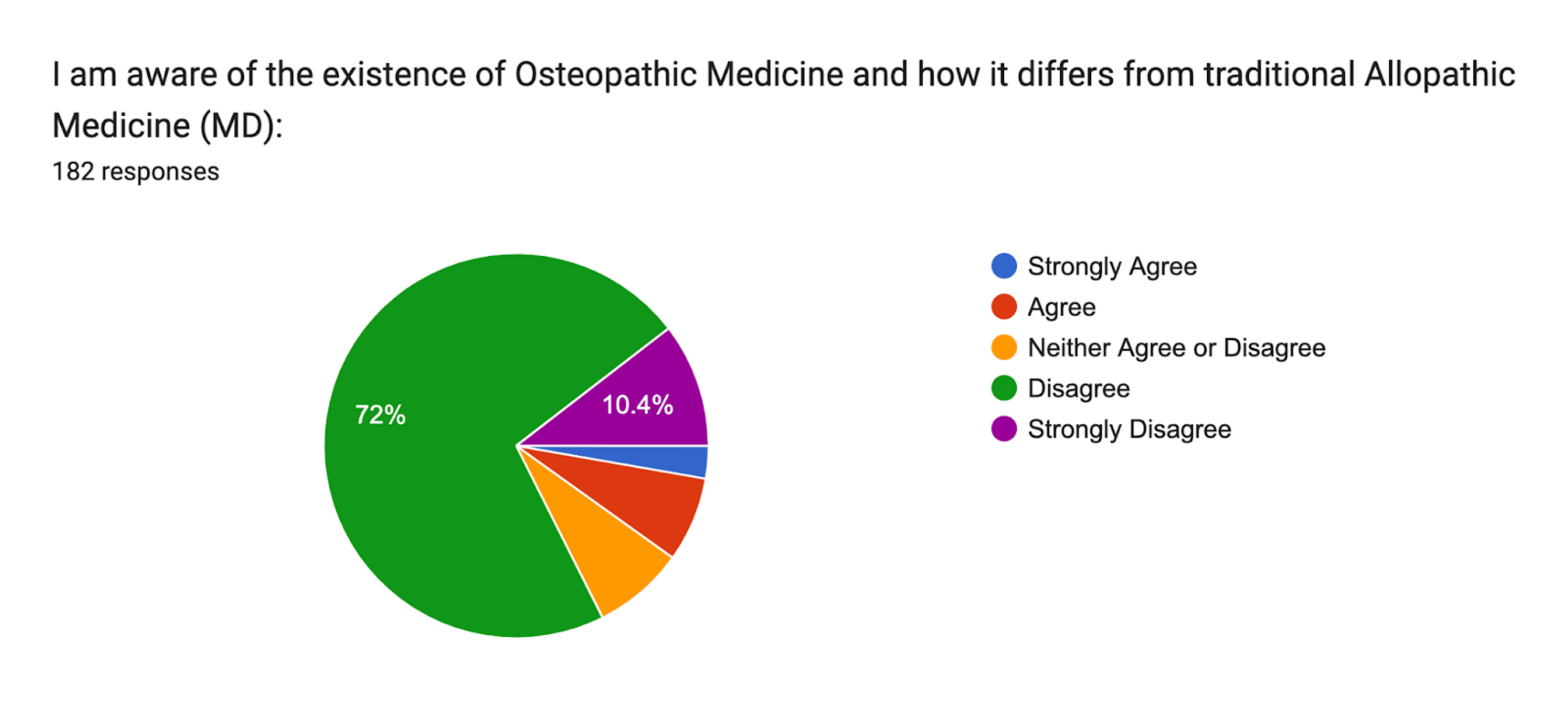
Awareness of Osteopathy Strongly Agree: n = 5, Agree: n = 13, Neither: n = 14, Disagree n = 131, Strongly Disagree n = 19

Awareness of osteopathic medicine and its distinction from allopathic medicine was reported as follows: 3% strongly agreed, 7% agreed, 8% were neutral, 72% disagreed, and 10% strongly disagreed (Figure 5).

**Figure 6:**
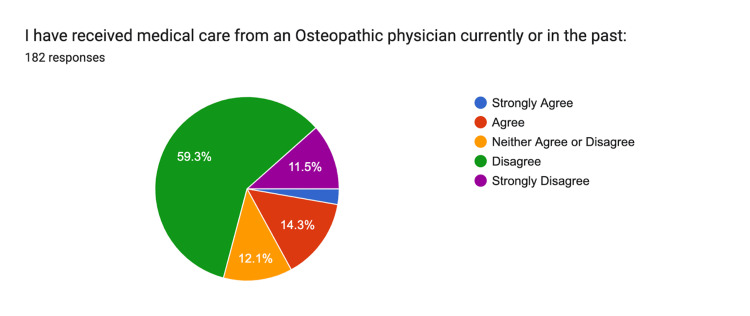
Medical Care Strongly Agree: n = 5, Agree: n = 26, Neither: n = 22, Disagree n = 108, Strongly Disagree n = 21

Regarding past medical care by an osteopathic physician, 3% strongly agreed, 14% agreed, 12% were neutral, 60% disagreed, and 12% strongly disagreed (Figure 6).

**Figure 7:**
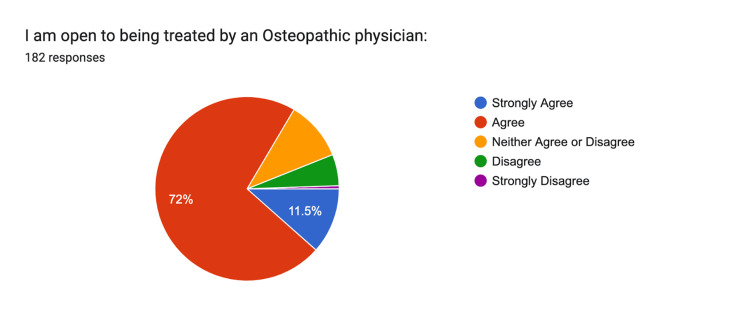
Openness to Treatment Strongly Agree: n = 21, Agree: n = 131, Neither: n = 19, Disagree n = 10, Strongly Disagree n = 1

Openness to treatment by an osteopathic physician showed with 72% agreeing and 12% strongly agreeing, with 6% disagreeing. A little over 10% remained neutral (Figure 7).

**Figure 8:**
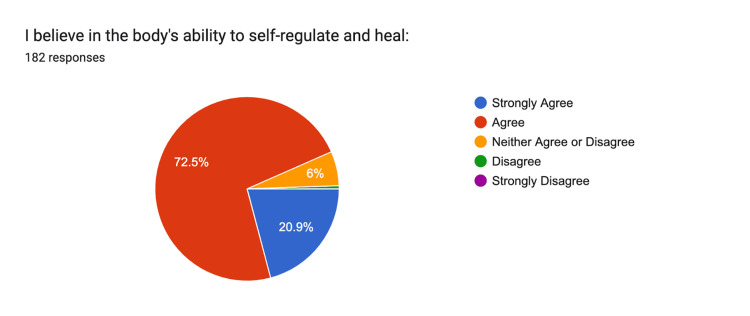
Self-Regulation Strongly Agree: n = 38, Agree: n = 132, Neither: n = 11, Disagree n = 1, Strongly Disagree n = 0

Belief in the body's ability to self-regulate and heal had 21% strongly agreeing and 73% agreeing (Figure 8).

**Figure 9:**
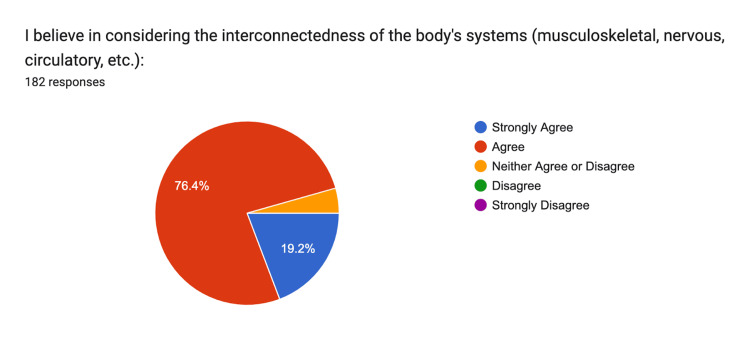
Interconnectedness Strongly Agree: n = 35, Agree: n = 139, Neither: n = 8, Disagree n = 0, Strongly Disagree n = 0

Similarly, 19% strongly agreed, and 76% agreed with the interconnectedness of the body's systems (Figure 9).

**Figure 10:**
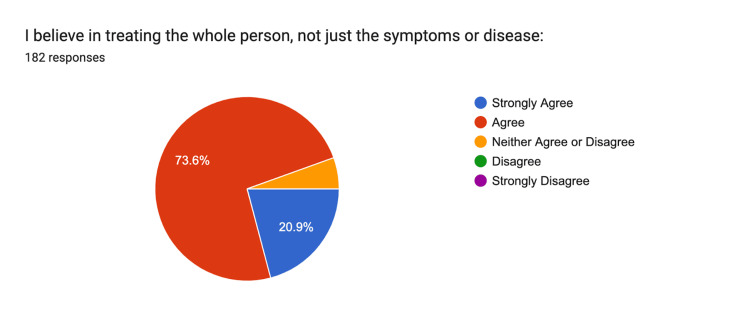
Treating the Whole Person Strongly Agree: n = 38, Agree: n = 134, Neither: n = 10, Disagree n = 0, Strongly Disagree n = 0

The holistic approach to medicine resulted in 21% strongly agreeing and 76% agreeing that treating the whole person, rather than just symptoms or disease, is essential (Figure 10).

**Figure 11:**
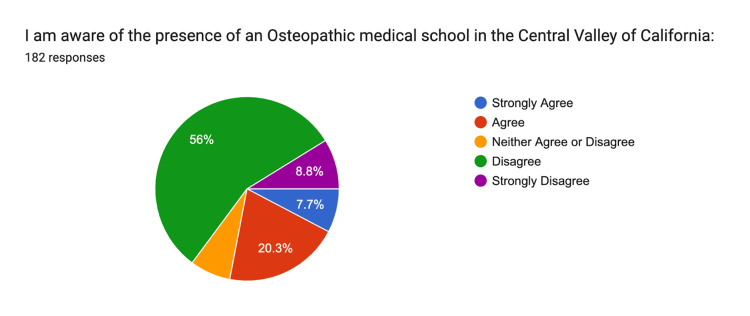
Osteopathic Medical School Awareness Strongly Agree: n = 14, Agree: n = 37, Neither: n = 13, Disagree n = 102, Strongly Disagree n = 16

Awareness of an osteopathic medical school in the Central Valley of California was reported with 8% strongly agreeing, 20% agreeing, 7% remaining neutral, 56% disagreeing, and 9% strongly disagreeing (Figure 11).

**Figure 12:**
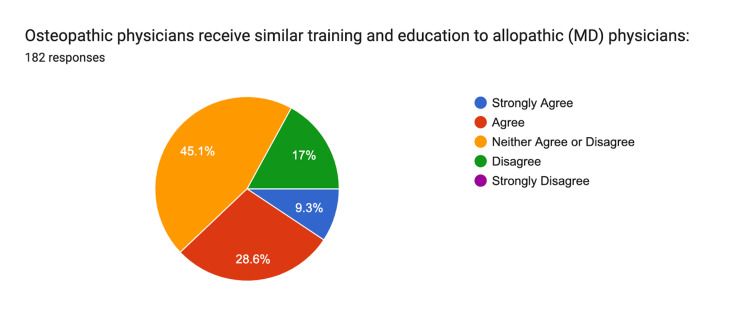
Similar Training Strongly Agree: n = 17, Agree: n = 52, Neither: n = 82, Disagree n = 31, Strongly Disagree n = 17

When asked whether osteopathic physicians receive similar training to allopathic physicians, 9% strongly agreed, 20% agreed, 45% were neutral, and 17% disagreed (Figure 12).

**Figure 13:**
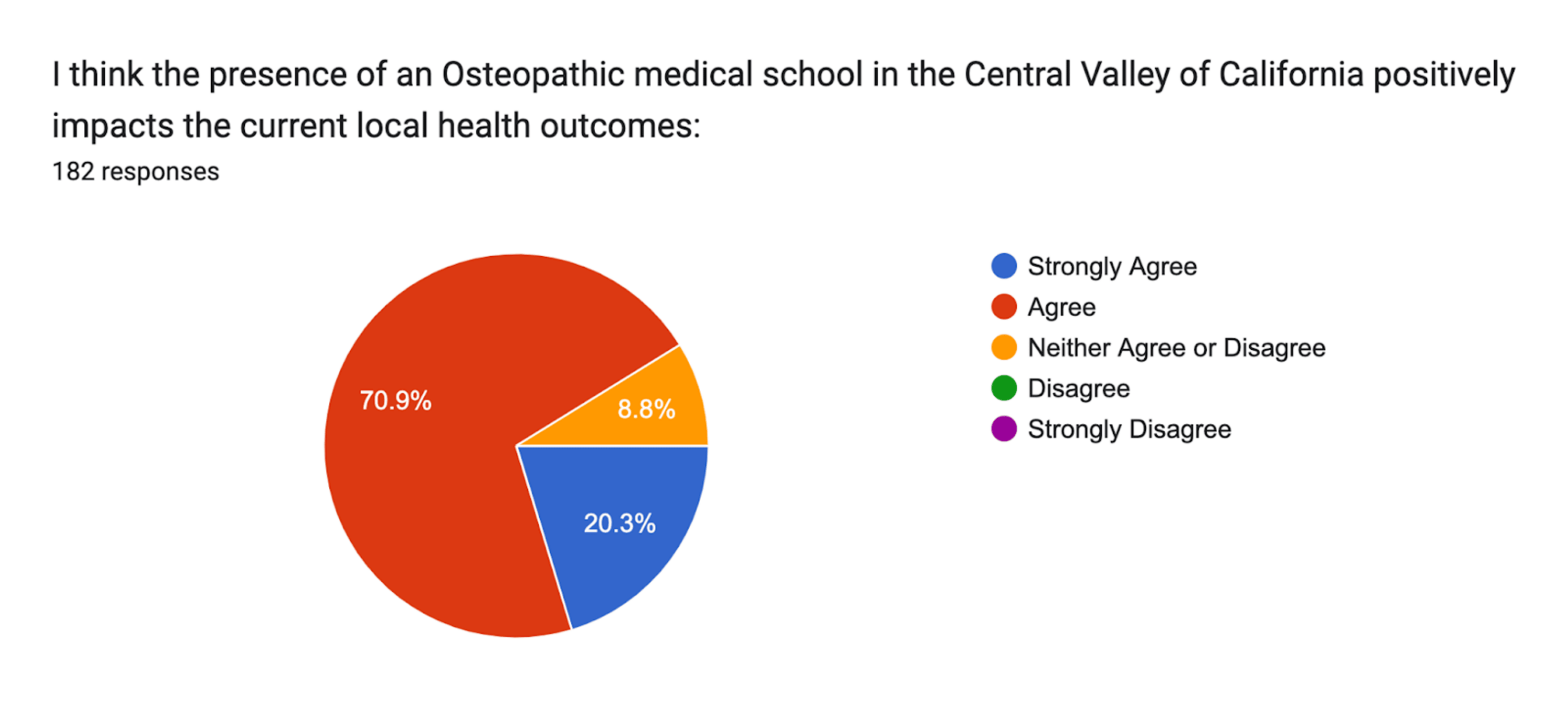
Positive Impacts Strongly Agree: n = 37, Agree: n = 129, Neither: n = 16, Disagree n = 0, Strongly Disagree n = 0

The perception of the positive impact of an osteopathic medical school in the region had 20% strongly agreeing, 71% agreeing, and 9% remaining neutral (Figure 13).

**Figure 14:**
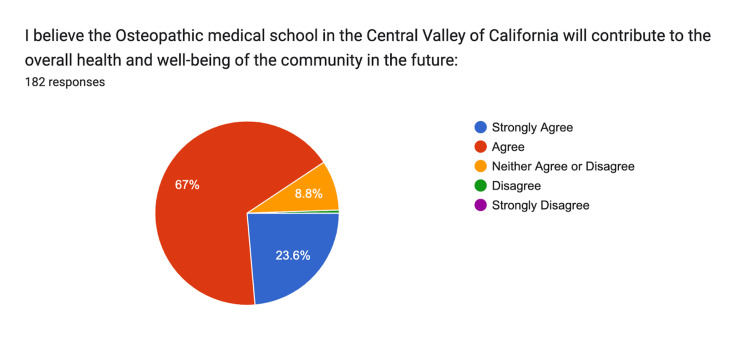
Community Health and Well-Being Strongly Agree: n = 43, Agree: n = 122, Neither: n = 16, Disagree n = 1, Strongly Disagree n = 0

Lastly, 20% strongly agreed, and 67% agreed that the osteopathic medical school would contribute to the overall health and well-being of the community in the future (Figure 14).

## Discussion

This study was conducted to assess the current level of community awareness of osteopathic medicine within Fresno County and to evaluate attitudes toward the potential impact of a new osteopathic medical school in the region. These findings reveal valuable insights into the level of awareness about osteopathic medicine and the community's openness to osteopathic treatments. In addition, this data can inform future educational and outreach efforts.

The survey results indicate a significant gap in public knowledge regarding osteopathic medicine, with many respondents (72%) unaware of its distinct philosophy and practices compared to allopathic medicine. Only 5% of respondents strongly agreed with understanding the differences between osteopathic and allopathic medicine, highlighting a clear need for targeted educational outreach. This lack of awareness is further underscored by 59% of participants who reported not receiving care from an osteopathic physician. These findings suggest that osteopathic medicine is not widely known or utilized in the region, likely due to the limited presence of osteopathic physicians and educational programs in Central California.

Despite the low awareness of osteopathy, there is a high degree of openness to osteopathic treatment. Over 80% of respondents expressed a willingness to receive care from an osteopathic physician, reflecting a positive receptivity to osteopathic care. This finding aligns with the belief held by 73% of participants in the body's ability to self-regulate and heal, principles at the heart of osteopathic philosophy. Moreover, 96% of respondents supported treating the whole person rather than focusing solely on symptoms or disease. While our survey found low awareness of osteopathic medicine but high openness to receiving osteopathic care, these results are descriptive and cannot establish causal relationships. The associations observed reflect potential opportunities for education and outreach rather than direct effects on healthcare behaviors. This result indicates an alignment with the holistic approach that osteopathy emphasizes.

Despite its limited awareness, these findings also highlight the community's openness to receiving osteopathic care. Over 80% of respondents were open to receiving treatment from an osteopathic physician, suggesting that while many are unfamiliar with osteopathy, they are receptive to its holistic, patient-centered approach. This openness, combined with the high level of agreement regarding the body's self-regulating abilities, presents an opportunity to build on these foundational beliefs and introduce osteopathic medicine as a viable treatment option. Public health campaigns and community outreach initiatives could leverage these existing attitudes to educate individuals on the benefits of osteopathic treatments and increase their utilization.

The American Association of Colleges of Osteopathic Medicine (AACOM) highlights that all top 14 U.S. medical schools for graduates entering primary care are osteopathic institutions, with many also ranking highly for rural practice, reinforcing that CHSU-COM's presence in Fresno could significantly address regional primary care needs and rural physician shortages [14]. The survey results strongly support the potential establishment of an osteopathic medical school in the region, with over 70% of respondents agreeing that such an institution would positively impact the local healthcare system. Although the survey indicates community support for a local osteopathic medical school, any anticipated impact of CHSU-COM on regional healthcare is speculative, as no intervention or longitudinal follow-up was conducted. This support highlights the community's recognition of the potential benefits of increasing access to osteopathic physicians. However, the low level of awareness regarding the existence of an osteopathic medical school in the Central Valley, with 56% of respondents disagreeing with its existence, points to a significant gap in disseminating information about educational initiatives in the region. Additionally, 45% of participants were neutral on whether osteopathic physicians receive similar training to allopathic physicians, indicating a lack of clarity on the distinctions between the two types of medical training.

Given the community's openness to osteopathic care and support for an osteopathic medical school, these findings underscore the need for targeted educational programs to raise awareness about osteopathic medicine and its unique approach. Institutions such as CHSU-COM can play a critical role in community outreach. Studies have shown that, by engaging directly with underrepresented groups, higher education institutions play a vital role in improving the well-being of their local communities [15]. Efforts can be further initiated to help bridge the knowledge gap through public health campaigns, workshops, and collaborations with local healthcare providers. These efforts should clarify the distinctions between osteopathic and allopathic medicine, emphasize osteopathy's holistic and patient-centered nature, and demonstrate its potential benefits in improving patient outcomes.

While this study provides valuable insights, several limitations are noted. The survey sample, although diverse, may not fully represent the entire Fresno County population. This is in rural areas where access to healthcare and educational resources may be limited. The study may be affected by selection bias due to the survey locations or the demographics of the respondents. In addition, outcomes in Fresno may be affected by the city’s cultural and linguistic diversity. Additionally, the findings may not be generalizable beyond Fresno County due to its unique demographics and healthcare context. Future research should include a broader sample size and explore the reasons behind the low level of awareness of osteopathic medicine using qualitative methods. Additionally, a longitudinal study could assess the effectiveness of educational interventions over time, helping to track changes in awareness and attitudes with educational initiatives.

While the community's awareness of osteopathic medicine is currently low, there is strong receptivity to its holistic principles and potential benefits. Establishing an osteopathic medical school in the Central Valley presents a promising opportunity to increase exposure to osteopathic care and integrate its principles into local healthcare. Targeted educational and outreach efforts are essential to bridging the knowledge gap, promoting osteopathic treatments, and ultimately improving the health outcomes of Fresno County residents.

## Conclusions

Our survey reveals a significant gap in community awareness of osteopathic medicine and its unique features and a general lack of familiarity with the potential impact of an osteopathic medical school in Fresno County. However, it also highlights a high degree of openness to osteopathic treatment and a strong belief in the potential benefits of an osteopathic medical school in this region. To address this lack of awareness, it is crucial to prioritize education and community engagement to elevate the recognition of osteopathy. With the addition of a new osteopathic medical school in California's Central Valley, CHSU-COM, there is an opportunity for more significant achievements and broader recognition of OMM through student community outreach. These findings underscore the need for targeted educational initiatives to increase awareness and engagement with osteopathic medicine, ultimately improving community health outcomes in the future. Further large-scale objective studies are necessary to draw conclusions, effectively gauge public awareness and understanding of osteopathic medicine, assess the community impact of emerging medical schools, and evaluate changes in awareness over time.
